# Potential Mechanisms and Effects of Efferocytosis in Atherosclerosis

**DOI:** 10.3389/fendo.2020.585285

**Published:** 2021-02-01

**Authors:** Lili Wang, Hongxia Li, Yuhan Tang, Ping Yao

**Affiliations:** ^1^Department of Nutrition and Food Hygiene, School of Public Health, Tongji Medical College, Huazhong University of Science and Technology, Wuhan, China; ^2^Hubei Key Laboratory of Food Nutrition and Safety, School of Public Health, Tongji Medical College, Huazhong University of Science and Technology, Wuhan, China; ^3^Ministry of Education Key Laboratory of Environment, School of Public Health, Tongji Medical College, Huazhong University of Science and Technology, Wuhan, China

**Keywords:** atherosclerosis, efferocytosis, macrophage, apoptosis, mechanism

## Abstract

Atherosclerosis (AS) is the main pathological basis for the development of cardio-cerebrovascular diseases. Abnormal accumulation of apoptotic and necrotic cells resulted in plaque enlargement, necrotic core formation and plaque rupture in AS. Under physiological conditions, apoptotic cells (ACs) could be effectively phagocytized and cleared by phagocyte-mediated efferocytosis. In contrast, the clearance efficiency of ACs in AS plaque was much lower because of the impaired efferocytosis in AS. Recent findings have made great progress on the molecular mechanisms of efferocytosis process and dynamic regulation, and its dysfunction on organismal health. Yet, there are still few effective treatments for this process. This article reviews the mechanism of efferocytosis and the role of efferocytosis in AS, highlighting a novel therapeutic strategy for AS, which mainly prevents the progression of plaque by targeting efferocytosis.

## Introduction

The human body turns over 100 billion cells every day to maintain normal development, tissue homeostasis, or physiological function by clearing unwanted (damaged, dysfunctional, aged, or harmful) cells. To remove these dying cells effectively, they are efficiently cleared by phagocytes in a process called programmed cell removal (PrCR), or efferocytosis (1, 2). Despite different types of cell death, the majority of what is known about efferocytosis involved the engulfment of caspase-driven apoptotic cells (ACs) initiated by intrinsic or extrinsic pathways, such as genotoxic stress or receptor-mediated death, respectively. Caspases activation induced a series of cellular changes such as DNA fragmentation, plasma membrane alteration, and the regulated release of cellular contents, facilitating efferocytosis by phagocytes (3). The phagocytes include professional phagocytes (such as macrophages and immature dendritic cells) and non-professional phagocytes (such as smooth muscle cells and endothelial cells with macrophage-like phenotype). As the main ones, macrophages play a critical role in identifying ACs for phagocytosis and clearance. In recent years, efferocytosis has attracted more and more attention on health and diseases. Dying cells could be removed by efferocytosis quickly in most cases, otherwise it would induce a break in tolerance to self-antigens and secondary necrosis, aggravating chronic inflammatory and autoimmune diseases, such as cardiovascular disease, rheumatoid arthritis (RA), systemic lupus erythematosus (SLE), and so on (4).

Atherosclerosis (AS) is a chronic progressive inflammatory disease that occurs in the arteries. Inflammation promoted the formation and development of plaques, which eventually led to plaque rupture (5). Importantly, impaired clearance of excessive ACs not only resulted in the accumulation of dying cells as main components of plaque, but also caused secondary necrosis and even life-threatening rupture in AS plaque (6). Considerable evidence suggested that efferocytosis was impaired in AS, which may be attributed to the dysfunction of phagocytes recognizing ACs, including damaged phagocytes themselves. Enhanced efferocytosis in AS can activate the clearance of ACs in plaque, reduce the occurrence of secondary necrosis, and improve plaque stability (7). However, the treatment of AS has not yet been satisfactory, especially under the circumstance of advanced AS. Some preliminary effects have been achieved on the treatment of efferocytosis-related diseases by focusing on signal molecules and regulating pathways, but the treatment of efferocytosis associated with AS is not refined because of their complicated interaction. To broaden the horizons, this paper summarizes the recent advances on efferocytosis pathophysiology in AS progression, and explores the underlying molecular mechanisms, including epigenetic regulation.

## Basic Efferocytosis Procedure

Efferocytosis is a highly conservative physiological process, involving the synergistic regulation of phagocytes and ACs. Effective clearance of ACs should be the ultimate destination of apoptosis, and it is also a key link to prevent inflammation and maintain tissue homeostasis under physiological conditions (8). Therefore, the body needs to accurately recognize and remove ACs to initiate and perform efferocytosis with the direction of a variety of signal molecules. These signals include: (1) “Find me” signal: Chemokines released from ACs induce phagocytes to the area of cell death; (2) Bridging molecules: Connecting phagocytes to ACs; (3) “Eat me” signal: As a ligand on the surface of ACs, it recognizes and binds to receptors of phagocytes through bridging molecules to induce efferocytosis; (4) “Don’t eat me”: Expressed in viable cells commonly, which can separate viable cells from ACs (9). As previously stated, joint regulation of these signal molecules determines whether the cell is cleared by phagocytes (**Table 1**).

**Table 1:** Signal molecules related to efferocytosis.

Role in efferocytosis	Molecule	Expression	Recognition receptor	Reference
Find me	LPC	Apoptotic cell	G2A	(10)
	CX3CL1	Apoptotic cell	CX3CR1	(11)
	S1P	Apoptotic cell	S1PRs	(12)
	ATP/UTP	Apoptotic cell	P2Y2	(8)
Eat me	PtdSer	Apoptotic cell	BAI1/TIM1/3/4/MFGE8-αVβ3	(13–16)
	Calr	Apoptotic cell	LRP1	(17)
	ICAM3	Apoptotic cell	CD14	(18)
Bridging molecule	MFGE8	Apoptotic/phagocytic cell	Integrin αvβ5/Integrin αvβ3	(13, 19)
	C1q	Phagocytic cell	SCARF1/Integrin αMβ2	(20, 21)
	GAS6	Apoptotic/phagocytic cell	AXL/Mertk	(22, 23)
	TSP-1	Apoptotic cell	CD36/Integrin αvβ3	(13, 24)
Don’t eat me	CD47	Viable cell	SIRPα	(25)
	CD31	Viable cell	CD31	(26)
	CD24	Viable cell	Siglec-10	(27)

LPC, Lysophosphatidylcholine; G2A, G-protein-coupled receptor; CX3CL1, CX3C chemokine ligand 1; CX3CR1, CX3C chemokine receptor 1; S1P, Sphingosine-1 phosphate; S1PRs, Sphingosine-1 phosphate receptors; ATP/UTP, Adenosine Triphosphate/Uridine Triphosphate; P2Y2, Purinergic receptor P2Y2; PtdSer, Phosphatidylserine; BAI1, Brain-specific angiogenesis inhibitor 1; Tim, T-cell immunoglobulin- and mucin-domain-containing molecule; MFGE8, Milk fat globule-EGF factor 8; Calr, Calreticulin; LRP1, Low-density lipoprotein receptor-related protein 1; ICAM3, Intercellular adhesion molecule 3; C1q, Complement 1q; SCARF1, Scavenger receptor F1; GAS6, Growth arrest-specific gene 6 product; AXL, Anexelekto receptor tyrosine kinase; Mertk, Mer tyrosine kinase; TSP-1, Thrombospondin-1; SIRPα, Signal regulatory protein α; Siglec-10, Sialic acid binding ig like lectin 10.

### Find Me

**Figure 1:**
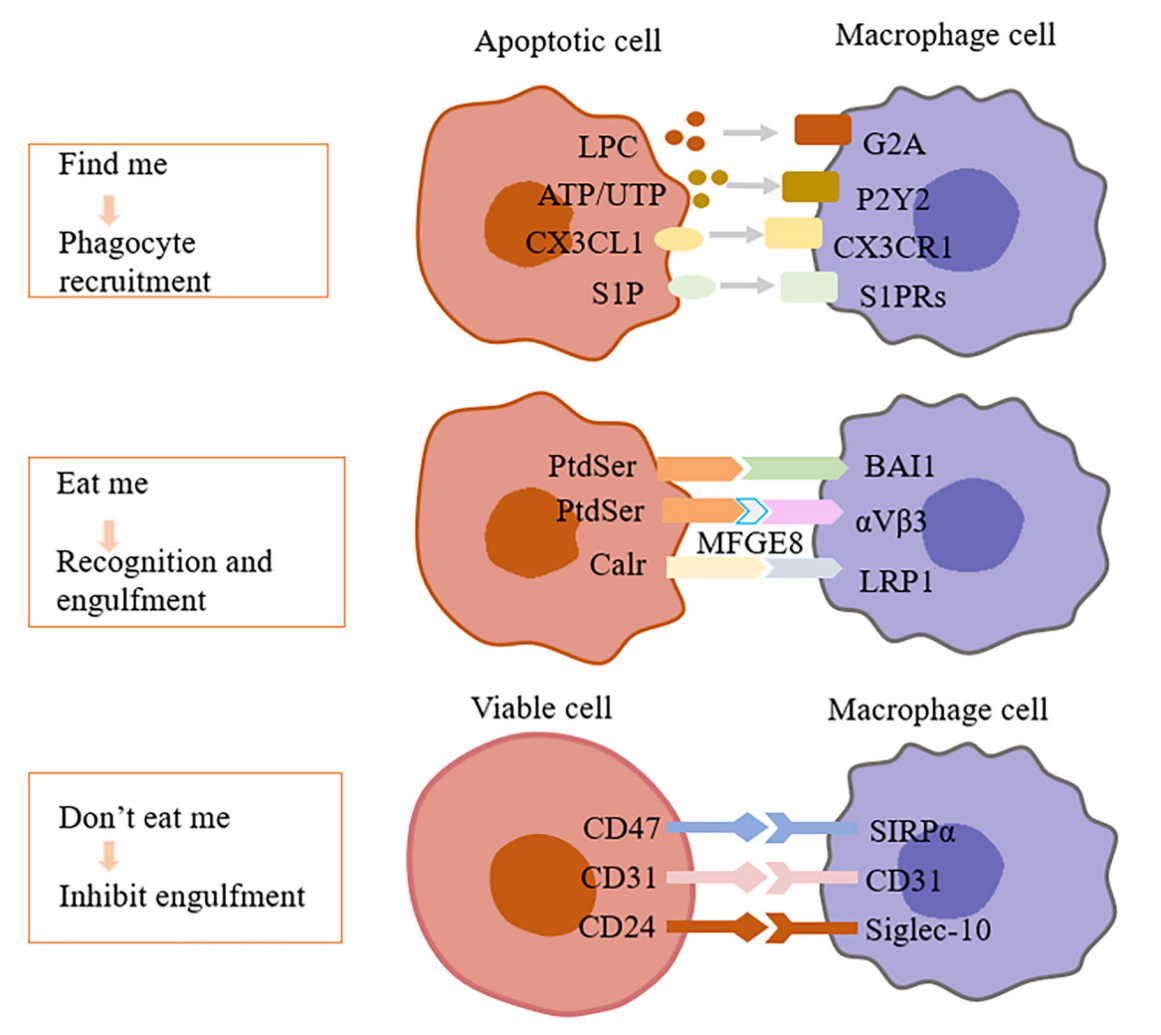
Interactions of efferocytosis signal molecules. Dying cells release “Find me” signals, such as lysophosphatidylcholine (LPC), ATP/UTP, fractalkine or S1P, recruiting phagocytes to sites of cell death. Phagocytes sense these “find-me” signals *via* cognate receptors (G2A, P2Y2, CX3CLR, and S1PRs, respectively). After that, dying cells expose a variety of signals on their surfaces, interacting with receptors on the phagocytic membrane through “Eat me” signals. The most common “Eat me” signal, phosphatidylserine (PtdSer), can interact with a variety of receptors on the surface of phagocytes, such as BAI1. In addition, PtdSer can facilitate uptake by bridging molecules such as MFG-E8, which engage other surface engulfment receptors (αvβ3). Other “Eat me” signals, such as calreticulin (Calr), mediate recognition and engulfment *via* the receptors LRP1. Healthy cells protect them from phagocytosis by expressing “Don’t eat me” signals, such as CD47, CD31, CD24, which binds to receptors (SIRP α, CD31, Siglec-10, respectively) expressed on phagocytes and inhibits the pathways needed for phagocytosis. LPC, Lysophosphatidylcholine; G2A, G-protein–coupled receptor; P2Y2, Purinergic receptor P2Y2; CX3CL1, CX3C chemokine ligand 1; CX3CLR, CX3C chemokine receptor 1; S1P, Sphingosine-1 phosphate; S1PRs, Sphingosine-1 phosphate receptors; PtdSer, Phosphatidylserine; BAI1, Brain-specific angiogenesis inhibitor 1; MFGE8, Milk fat globule-EGF factor 8; Calr, Calreticulin; LRP1, Low-density lipoprotein receptor-related protein 1; SIRP α, Signal regulatory protein α; Siglec-10, Sialic acid binding Ig like lectin 10.

Before the phagocytosis, the crucial step for ACs is to “be found” by phagocytes from the sea of living cells, which is a fascinating question worth to be explored in depth. Emerging studies showed that ACs released various mediators or termed “Find me” signal molecules (3). These “Find me” signals mainly include lysophosphatidylcholine (LPC), CX3C chemokine ligand 1 (CX3CL1), sphingosine-1-phosphate (S1P), nucleotides ATP, UTP, etc. (**Figure 1**) (8). In addition to recruiting phagocytes, of note, some “Find me” factors contain several extracellular signaling peptide chain with potential capabilities to modify and prepare the microenvironment for cell clearance (3).

As the first identified and main form of “Find me” signal, LPC, is released from ACs through hydrolyzing membrane phosphatidylcholine by caspase-3-activating Ca^2+^-independent phospholipase A2 (IPLA2). However, high serum LPC was shown to link with the accumulation of dead cells in blood, skin, and lymph nodes of patients with SLE. Excessive LPC spilled from efferocytosis-resistant tissues might interfere with normal ACs “Find me” signal by lowering LPC concentration gradient between dying cells and phagocytes, which was presumed as a cause as well as a consequence of SLE (28). The ATP-binding cassette transporter A1 (ABCA1) is essential for lipid transport and metabolism, which promotes cholesterol efflux to lipid-poor APOA1, the building block of HDL, and it is an evolutionarily conserved gene associated with AC clearance (29). Peter et al. (29) found that ABCA1 might be responsible for LPC release. By binding with G-protein-coupled receptor G2A, ACs-released LPC did induce the chemotaxis of phagocytes (**Figure 1**). Interestingly, other lysophospholipids hydrolyzed by IPLA2 or metabolic derivatives of LPC failed to exert LPC-like activity, and even neutralized LPC role in a G2A-dependent manner (10). Nevertheless, Murakami et al. (30) reported that LPC suppressed G2A-mediated actin polymerization, which raises a dispute about whether G2A is a target receptor of LPC. G2A knockdown did not completely inhibit phagocytes migration towards ACs (10), indicating at least that other targets of LPC may exist for further research (**Figure 1**).

### Eat Me

“Eat me” ligands on ACs interact with receptors on phagocytes, by which macrophages find ACs under the action of chemokines to trigger efferocytosis (31). Receptors on phagocytes include but not limit to Mer receptor tyrosine kinase (Mertk), low-density lipoprotein (LDL) receptor-related protein 1 (LRP1), scavenger receptor class B type I (SR-BI), T-cell immunoglobulin- and mucin-domain-containing molecule (Tim) -1/Tim-4 (15, 32, 33). The “Eat me” signal mainly includes phosphatidylserine (PtdSer), calreticulin (Calr) and intercellular adhesion molecule 3 (ICAM3). Among them, PtdSer is the key eating signal, which is located on the inner surface of the plasma membrane under physiological conditions. However, PtdSer is reversed to the outer surface of the plasma membrane under the action of phospholipid transferase in ACs to bind to the receptor of phagocyte (15) (**Figure 1**). This is explained by the fact that the role of the ATP-dependent ATP enzyme ATP11 restricts PtdSer to the inner leaflet of the plasma membrane under normal circumstances. In the process of apoptosis, caspase3 cleavage inactivates ATP11 and promotes the exposure of PtdSer on the surface of ACs (34). Thus, the lack of such signals could lead to the accumulation of ACs and drive the occurrence of AS. However, although PS externalization is one of the landmarks of effective efferocytosis of dying cells, PS is also externalized on some living cells. Interestingly, phagocytes can distinguish between living and ACs exposed to PS, so stimulation of living cells does not induce phagocytosis (35).

### Bridging Molecules

Bridging molecules are the key factors in the phagocytic ability of phagocytes. Extracellular bridging molecules include milk fat globular epidermal growth factor 8 (MFGE8), serum complement 1q (C1q), growth arrest-specific gene 6 product (GAS6), thrombospondin-1 (TSP-1), etc (19–24). MFGE8 is a kind of lactadherin, which is mainly secreted by activated macrophages. One side of MFGE8 is connected with PtdSer on the surface of ACs, and the other side is connected with integrin αvβ3/αvβ5, which is like a bridge between ACs and macrophages, promoting efferocytosis (13, 19) (**Figure 1**). In the mouse model, decreased expression of MFGE8 was observed in Alzheimer’s disease (AD). AD is a degenerative disease of the central nervous system, and neuronal apoptosis is an important part of the pathogenesis of AD (36). Besides, the decreased expression of MFGE8 in the plaque of advanced AS affected the clearance of ACs. However, the increased expression of MFGE8 protein in the tumor environment not only increased the efferocytosis, but also promoted the initiation, development and metastasis of tumor. It is because the interaction between MFGE8 apoptotic cancer cells and macrophages activated STAT3 phosphorylation and induced macrophages to polarize into the M2 phenotype. In cancer, M2 macrophages as tumor-associated macrophages have anti-inflammatory and tumor-promoting properties (37).

### Don’t Eat Me

Non-apoptotic cells send a “Don’t eat me” signal, such as CD31, CD46, CD47 (38), to avoid efferocytosis when exposed to phagocytes. The most common ligand is CD47, which binds to the phagocytic inhibitory receptor signal regulatory protein α (SIRPα) on phagocytes to avoid phagocytosis. A few “Eat me” signals are expressed in viable cells, such as Calr and PtdSer. Interestingly, the presence of CD47 could trigger the SIRPα receptor to resist the efferocytosis and prevent viable cells from being cleared (25). Moreover, the level of CD47 decreased in a caspase-dependent manner during apoptosis, which seemed to be the result of the active shedding of these proteins in the released particles (39). In addition, a recent study revealed that CD24, a new “Don’t eat me” signal, interacted with Sialic acid Ig like lectin 10 (Siglec-10) to prevent macrophages from clearing cancer cells (27) (**Figure 1**). How to destroy the active phagocytosis inhibition of diseased cells so as to promote the absorption of diseased cells remains to be further studied.

### Post Engulfment Stage

Macrophages phagocytize ACs by rearranging the cytoskeleton to form phagocytic cups, and activating Rac1 mediated actin remodeling to promote engulfment (40). Phagocytes formed a large vacuole containing dead cells after phagocytosis of ACs, and then lipids, proteins and nucleic acids derived from ACs accumulated in a vacuole. Normally, macrophages prevented these substances from accumulating in cells through self-digestion, degradation, and efflux pathway. For instance, PS on the surface of ACs upregulated the expression of ABCA1 in macrophages to promote cholesterol efflux to extracellular cholesterol receptors (especially apolipoprotein A1) (41). Apparently, if these phagocytes successfully regulated the process of phagocytosis and digestion in AS plaques, then it was likely to result in the inhibition of foam cell formation and maintenance of tolerance to autoantigens. In addition, Yurdagul et al. (42) recently showed that macrophages assimilated arginine and ornithine from ACs and converted to putrescine through arginase 1 (Arg1) and ornithine decarboxylase (ODC) in the process of efferocytosis, and putrescine enhanced subsequent efferocytosis by activating Rac1. Therefore, macrophages reprogramming and digested molecules could provide additional energy for phagocytosis after phagocytic ACs, which was essential to ensure subsequent phagocytosis and immune response of phagocytes.

## Atherosclerosis Pathophysiology

AS is a progressive disease that occurs in the intima of large and medium-sized arteries, which is the main pathological basis of cardio-cerebrovascular diseases. Disorder of lipid metabolism, chronic inflammation and abnormal immune response are the characteristics of AS. During its development, the accumulation of ACs plays an important role in the formation and stability of plaques (6, 43).

Nowadays, it is common that the concentration of cholesterol in the blood exceeds the biological needs of organisms, which leads to the development of AS, whereas how excessive LDL-C leads to AS is unclear (43). LDL-C can be catalyzed by metal ions (Fenton reaction) to form reactive oxygen species in the intima. Particularly, cardiovascular phagocytes bind lipoprotein particles to promote the formation of foam cells through receptors of the LRP superfamily. Initially, the formation of foam cells is beneficial because it isolates potentially destructive lipoproteins. However, it gradually exceeds the ability of foam cells to process lipoproteins over time, which leads to endoplasmic reticulum (ER) stress to produce ROS and trigger apoptosis (44). Ultimately, foam cells release their contents into the extracellular space, thus worsening the inflammatory state and stability of plaques.

Atherosclerotic plaques develop through the continuous accumulation of lipids and lipid-filled cells. In addition to LDL, other risk factors related to the formation of AS include hypertension, smoking and metabolic syndrome. Until now, the relationship between these factors and the formation of AS has not been fully elucidated (45). However, there is evidence that many risk factors are involved in inflammatory activation, which can alter the function of arterial wall cells. Much research on the mechanism of AS have demonstrated that modified lipids made macrophages and T cells show continuous inflammatory response and release inflammatory mediators. Furthermore, macrophages that exhibit anti-inflammatory properties seem to reduce AS, such as M2 macrophages. M2 macrophages have higher efferocytosis ability than M1 macrophages (46). M2 macrophages are polarized by Th2 cytokines such as IL-4 and IL-13 and secrete anti-inflammatory cytokines such as IL-10 and TGF-β to inhibit the recruitment of inflammatory cells (47). In addition, M2 macrophages express high levels of Arginase 1 and have increased secretion collagen, which could promote tissue repair (48). Different macrophage populations have different functions, such as M1, M2, or Mox macrophages. Recent studies based on unbiased single-cell RNA sequencing analysis have identified transcriptional markers of aortic macrophage populations and monocyte-derived dendritic cells in AS, and the use of these genes may allow further exploration and targeting of different macrophage and dendritic cell populations and their functions in AS (49). Altogether, the complex inflammatory process leads to the slow progression of AS lesions and is difficult to cure. The regression of inflammation helps to restore homeostasis in the body. In advanced AS, unresolved inflammation promotes persistent plaque inflammation, and large necrotic cores drive the development of AS lesions (50).

The experimental data showed that the metaplasia of smooth muscle cells (SMCs) might produce foam cells, which is similar to macrophages. Besides, SMCs and macrophages proliferate and produce extracellular matrix to form atherosclerotic plaques (51). With the progress of the lesion, SMCs produce matrix metalloproteinases (MMP) to degrade arterial extracellular matrix, and activated inflammatory cells secrete cytokines that inhibit the formation of fibrous caps and proteases that degrade fibrous caps. Furthermore, given that the inflammatory process hinders SMCs in AS plaques from synthesizing interstitial collagen and weakens the ability of cells to maintain fibrous cap skeletons, which is easy to make AS plaques ruptured with thrombosis (52).

Apoptosis can occur in many cell types, such as endothelial cells, SMCs, macrophages and T lymphocytes. Among them, macrophages account for more than 40% of dead cells (53). Normally, ACs are identified and targeted by phagocytes, and it is almost improbable to identify TUNEL-positive apoptotic bodies in healthy tissues. Thus, the detection of co-localization of apoptotic bodies and phagocytes indicated the existence of efferocytosis (54). The “free” apoptotic bodies could be detected in lesions, and necrotic core and fibrous cap were found in the progressive plaques, suggesting an impaired efferocytosis.

## Efferocytosis Impairment in AS Progression and Potential Mechanisms

The dysfunction of the efferocytosis during the development of AS results in the inability of ACs to be removed in time. It is plausible that defective efferocytosis is associated with the impairment of the phagocytic efficiency of phagocytes and the reduced edibility of ACs in plaques (55).

### Decreased Phagocytic Efficiency of Phagocytes

Several studies have suggested that the working efficiency of phagocytes has decreased, which may be due to a decrease in the absolute number of phagocytes or a decrease in phagocytic ability in atherosclerotic plaques. First, ER stress mediated vascular inflammatory responses and endothelial dysfunction during AS development, induced macrophage and vascular smooth muscle cell apoptosis, and loses the phagocytic capacity (44). In addition, the increase of phagocytes with lower phagocytic capacity may reduce phagocytic efficiency in AS. The polarization of macrophages was more inclined to pro-inflammatory M1 phenotype, which reduced the proportion of M2 phenotype with high phagocytic activity (56); SMCs lost their phagocytic capacity after contact with oxidized lipids (57); the phagocytic capacity of dendritic cells was also decreased in AS (32).

**Figure 2:**
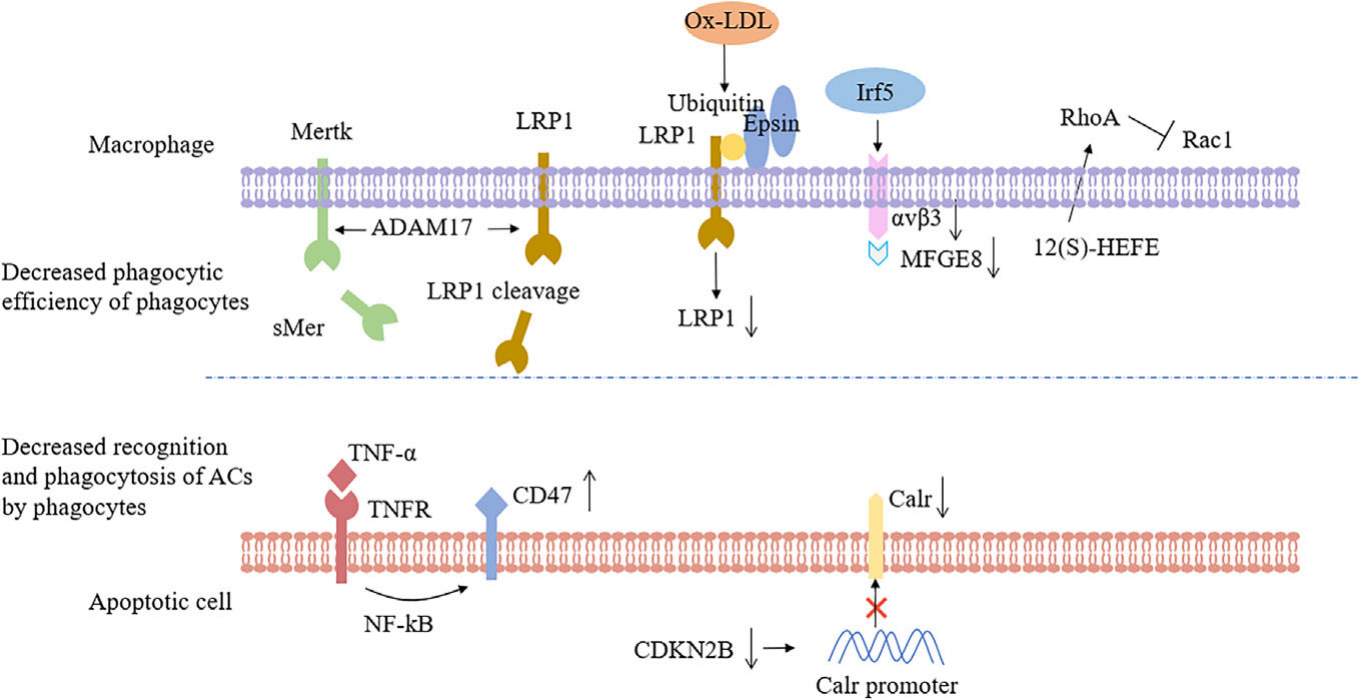
Impaired efferocytosis occurs as a result of poor phagocytic efficiency or decreased ability of apoptotic cells to be recognized and phagocytized. In atherosclerosis, macrophage recognition and phagocytosis processes were disturbed, such as Mertk or LR1 cleavage, ox-LDL internalization, and down-regulation of LRP1 in response to epsins, down-regulation of αvβ3 and MFGE8 by the transcription factor Irf5, and activation of RhoA by 12(S)-HEFE, which resulted in decreased phagocytic capacity. Upregulation of CD47 on the surface of apoptotic cells or inhibition of Calr expression by reduced CDKN2B levels would make apoptotic cells not bind to adjacent phagocytes. Mertk, Mer tyrosine kinase; sMer, soluble MER; LRP1, Low-density lipoprotein receptor-related protein 1; ADAM17, A disintegrin and metalloprotease 17; ox-LDL, oxidized low density lipoprotein; Irf5, Interferon regulatory factor 5; MFGE8, Milk fat globule-EGF factor 8; 12(S)-HEFE, 12(S)-hydroxyeicosate-traenoic acid; RhoA, Ras homolog gene family, member A; Rac1, Ras-related C3 botulinum toxin substrate 1; TNF-α, Tumor necrosis factor-α; NF-kB, nuclear factor-k-gene binding; CDKN2B, Cyclin-dependent kinase inhibitor 2B; Calr, Calreticulin.

Secondly, some studies have shown that receptors on the surface of macrophages could be shed during AS. Indeed, the loss of phagocyte receptors, such as Mertk, CD36 and LRP-1, resulted in the inability to remove ACs. Garbin et al. (58) indicated that soluble Mertk existed in AS. In the AS plaques, the expression of matrix metalloproteinases of various types was elevated, and a disintegrin and metalloprotease 17 (ADAM17) has been identified as the cause of the absence of Mertk and LRP1 (59, 60). In addition to Mertk, loss of macrophage LRP1 expression during AS progression led to accelerate plaque growth because of enhanced macrophage apoptosis by inhibiting protein kinase B activation (61). Besides, as a receptor for “Eat me” signaling, deficiency of LRP1 reduced efferocytosis, and caused macrophages to upregulate secretion of the inflammatory cytokines, such as tumor necrosis factor-α (TNF-α), monocyte chemoattractant protein-1, and MMP-9. Brophy et al. (62) found that oxidized low-density lipoprotein (ox-LDL) treatment increased the ubiquitination of LRP-1, followed by binding to epsin, and internalization from the cell surface, suggesting that epsins promote ubiquitin-dependent internalization and downregulation of LRP-1 (**Figure 2**). Nevertheless, Mueller et al. (63) found that mice lacking LRP1 macrophages expressed fewer M1 macrophages during plaque regression and promoted resolution of AS, so loss of LRP1 had a dual and opposite effect on plaque biogenesis.

Thirdly, more and more evidence suggested that non-coding RNA was associated with impaired efferocytosis and participates in epigenetic regulation by regulating gene expression. Dysregulation of the expression and function of microRNAs (miRs), non-coding RNAs involved in the post-transcriptional regulation of gene expression, presumably caused efferocytosis defects. Indeed, MiR-155 has been shown to inhibit macrophage-mediated efferocytosis and enhance foam cell aggregation in atherosclerotic lesions by inhibiting the expression of bcl6 (64). Bcl6 is an effective transcriptional inhibitor, which is highly expressed in advanced AS lesions. It may indirectly block the activation of RhoA (Ras homolog gene family, member A), RhoA is a small GTPase, that regulates multiple cellular processes involving the actin cytoskeleton (65). Hence, the inhibition of Bcl6 by miR155 led to the overactivation of RhoA, which has a negative effect on the cytoskeleton remodeling of macrophages and damages the efferocytosis.

Finally, the latest research has found many molecules related to defective efferocytosis in AS, which led to the decrease of phagocytosis of macrophages. Seneviratne et al. (66) detected the elevated expression of interferon regulatory factor 5 (Irf5) at arterial root lesions in mice and demonstrated that the transcriptional regulator Irf5 impaired efferocytosis by decreasing expression of MFGE8 and Itgb3 (the αvβ3 receptor with α5 integrin). Manega et al. (67) suggested that 12-HETE levels were higher in CAD patients, and monocyte-derived macrophages treated with 12(S)-HEFE had decreased efferocytosis because of increased activated forms of RhoA (**Figure 2**). Fredman et al. (68) have shown that the production defect of specialized pro-resolving mediators (resolvin D1) in late human plaques can damage the efferocytosis, and promoting the resolution of inflammation can increase the phagocytic ability of macrophages. In brief, the above results clearly manifested that the number of phagocytes and the phagocytic capacity decreased in atherosclerotic plaques.

### Decreased Recognition and Phagocytosis of ACs by Phagocytes

Compared with the other tissues of the body, the ability of ACs to be recognized and phagocytized by phagocytes decreased significantly in plaques. Given that the diseased tissue is in a state of chronic inflammation during the development of AS, and the inflammatory factor TNF-α could up-regulate the expression of CD47 molecules. While the CD47-mediated “Don’t eat me” signal was enhanced, which made the ACs could not be cleared in time, and the efferocytosis ability decreased (7) (**Figure 2**). Ye et al. (69) recently found that myocardial infarction-associated transcripts (MIAT), a highly conserved mammalian lncRNA, was significantly up-regulated in patients with atherosclerotic vulnerable plaques and continued to increase in the serum and macrophages of necrotic cores. MIAT acts as a miR sponge to positively regulates the expression of CD47 by sponging miR-149-5p. Moreover, HMGB1, an exocrine pro-inflammatory signal molecule, simultaneously acted on integrin αvβ3 and PS, and blocked the efferocytosis signal pathway during AS significantly (70).

As the lesion progresses, more ROS is produced in macrophages, leading to LDL oxidation. Ox-LDL competes with ACs and interacted with receptors of phagocytes (i.e. SR-BI) to weaken efferocytosis (71). Interestingly, continuous infusion of LPC (a major component of oxidized low-density lipoprotein) into hypercholesterolemic mice impaired the clearance of ACs by interfering with the LPC signaling gradient, which is required for the recruitment of phagocytes to dead cells (72). Furthermore, ox-LDL increased the expression of toll-like receptor-4 (TLR4), which may lead to an increase in the secretion of pro-inflammatory cytokines (TNF-α, IL-1β) and a decrease in the production of anti-inflammatory cytokines (TGF-β, IL-10). More than that, TLR4 reduced the expression of SR-BI and LRP1, while the increase in inflammatory cytokines reduced the activation of Liver X Receptor (LXR), which could promote the reduction of Mertk expression (73).

It has been revealed that the genotypes of the patients are susceptible to AS leading to impaired efferocytosis. Genome-wide analysis of patients with hereditary coronary atherosclerosis indicated that the 9p21.3 allele variant was associated with AS burden, and loss of cyclin-dependent kinase inhibitor 2B (CDKN2B) at this locus promotes vascular SMC apoptosis and aneurysm progression (74). Subsequently, Kojima et al. (75) demonstrated that decreased expression of CDKN2B could decrease the expression level of Calr, an important “Eat me” signaling molecule, which would lead to the escape of a large number of apoptotic SMCs from the efferocytosis and then cause atheromatous plaque enlargement (**Figure 2**).

Finally, although there are more ACs in the plaque than in normal tissue, the impaired efferocytosis has nothing to do with the increase of ACs. A large number of experiments have shown that the phagocytic capacity of macrophages under normal circumstances was sufficient to clear ACs completely (76). Together, the causes of efferocytosis impairment in AS plaques are mainly due to a decrease in the capacity of phagocytosis and imbalance in the pro-phagocytosis and anti-phagocytosis signatures on target cells, and other factors such as genetics, immunity, and inflammatory reactions are also worthy of further research.

## AS Deterioration by Impaired Efferocytosis and Potential Mechanisms

Atherosclerotic lesions could gradually impair efferocytosis, while the defective efferocytosis caused lipid accumulation, secondary necrosis, subsequent inflammatory responses, and autoimmune responses (76). Importantly, the accumulation of ACs resulting from impaired efferocytosis directly contributed to the formation of atherosclerotic plaques and necrotic lipid cores. Thus, a vicious cycle was formed between the progression of AS lesions and the impairment of efferocytosis.

Firstly, impaired efferocytosis may promote the progression of AS by increasing the release of inflammatory cytokines. When dead cells were successfully removed by, anti-inflammatory factors were subsequently produced. Therefore, efferocytosis defects decreased anti-inflammatory response, and the persistent tissue injury and damage-associated molecular patterns (DAMPS) mediated inflammation increased. Fadok et al. (77) was first demonstrated that phagocytosis of macrophages released beneficial factors such as TGF-β1, prostaglandin e2, and platelet-activating factor, which inhibited the production of pro-inflammatory cytokines through autocrine/paracrine action and reduced the aggregation of monocytes to inflammatory areas. Then, TGF-β1 was also found to be increased after efferocytosis of bone marrow stromal cells (78). Following apoptotic cell engulfment, macrophages activate tolerogenic pathways to prevent immune responses, while anti-inflammatory factor production after efferocytosis presumably depends on the type and activation state of phagocytes. Moreover, SR-BI^-/-^ macrophages impaired efferocytosis of ACs in atherosclerotic lesions, eliciting inflammatory response (higher interleukin IL-1β, IL-6, and TNF-α, lower IL-10, and TGF-β1), and necrosis (33). Therefore, in the absence of effective efferocytosis molecules, such as SR-BI, LRP1, and Mertk, ACs could not be eliminated in time, increasing the production of inflammatory factors and promoting the formation of necrotic core. Thorp et al (79). found that mutations in phagocytic Mertk receptors inhibited efferocytosis and accelerated the formation of necrotic plaques in ApoE^-/-^ mice. What is more, GAS6 deficiency promoted the development of AS by affecting the binding of Mertk and PS to reduce the recruitment of phagocytes in the lesions. Whereas GAS6 was mainly produced by SMCs, the apoptotic SMCs increased and GAS6 synthesis decreased in the progressive plaques, thus forming malignant circulatory lesions (80).

**Figure 3:**
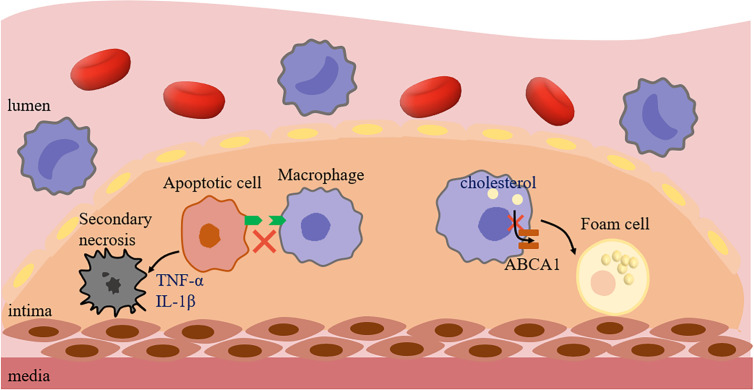
Impaired efferocytosis promotes AS. The lack of signal molecules in ACs caused macrophages to not effectively recognize and phagocytize ACs, and then caused ACs to release inflammatory factors, such as TNF-α and IL-1β, resulting in secondary necrosis, promoting the formation of necrotic core and leading to plaque instability. In addition, if the Calreticulin expression level of ACs around macrophages decreased, followed by failure efferocytosis and decreased ABCA1 expression, and macrophages were more likely to show foam cell phenotype. ABCA1, ATP-Binding Cassette Transporter 1; TNF-α, Tumor necrosis factor-α; IL-1β, Interleukin 1β.

Furthermore, foam cells have been well-documented as an important initiating factor in the development of AS. Importantly, impaired efferocytosis might lead to the transformation of more macrophages into foam cells. In the normal process of efferocytosis, phagocytes can reverse transport the ingested lipids to the extracellular environment after phagocytosis, thereby maintaining the normal function of phagocytes and continuing to play the role of phagocytosis (41). Kojima et al. (75) suggested that the phenotype of ACs had a significant effect on the behavior of nearby macrophages and their ultimate ability to maintain lipid homeostasis. Indeed, the impaired efferocytosis signal caused by the deficient expression of SMCs calreticulin may down-regulate the expression of ABCA1 in neighboring macrophages to inhibit cholesterol efflux in AS, resulting in phagocytes tending to foam cell phenotype, which may be due to the inability to activate Calr receptor LRP1/cholesterol efflux pathway. And then, the formation of foam cells could lead to cytotoxicity through inflammation and oxidative stress of endoplasmic reticulum. This defect would help to accumulate potentially toxic components in macrophages, such as unesterified cholesterol, and accelerates the formation of AS (**Figure 3**).

Additionally, in addition to ACs, cells with non-apoptotic programmed cell death could not be cleared in time, which also promotes the development of AS lesions. Gerlach et al. (81) found that the accumulation of necroptotic cells (NCs) in plaques was related to impaired efferocytosis, which was due to the expression of CD47 on NCs. Similarly, the accumulation of NCs would lead to the formation of larger necrotic cores in plaques and the more prone to rupture. Indeed, both apoptotic and non-apoptotic cells (such as apoptosis, necroptosis, pyroptosis, and ferroptosis) could release recognition molecules that guide subsequent phagocytic processes (82). However, the environment due to oxidation or inflammation in AS may result in the inability of dying cells to be efficiently removed, because many studies have found that the expression of CD47 on cells is affected by inflammatory factors, such as TNF-α, IL-1β, IL-6 (7, 83, 84). As expected, defective efferocytosis is an important link to promote AS, which not only increases the formation of foam cells, but also plays an irreplaceable role in the formation, enlargement, and rupture of plaques.

## Breakage of Vicious Circle by Efferocytosis Induction for AS Treatment

Efferocytosis as a target for diagnosis and treatment of AS has great potential. Currently, the mechanism for the treatment of AS by promoting efferocytosis is mainly to improve the phagocytosis and increase the secretion of molecules that promote efferocytosis (85). It may be feasible that the transformation of macrophages to the anti-inflammatory M2 phenotype improved the phagocytic capacity. Therefore, targeting the polarization of macrophages to modulators of the M2 phenotype may be a promising therapeutic strategy for AS. Bories et al. (86) found that nagilactone B, a specific agonist of LXR, can promote M2 polarization and efferocytosis of macrophages, reduce plaque formation and the area of necrotic core; Oh et al. (87) showed that the absence of vitamin D receptor (VDR) increased AS by making lipid-rich M2 monocytes adhere, migrate, and carry cholesterol into AS plaques, as well as by increasing macrophage cholesterol uptake and esterification. While VDR deletion was an extreme case of VD deficiency and may not directly represent the physiology of animals and humans, experiments have shown that adequate dietary VD affects M2 polarization processes. Bi et al. (88) summarized several potential targets and compounds for promoting M2 polarization as potential therapeutic targets for AS, such as inhibiting or activating certain enzymes (dipeptidyl peptidase), affecting transcription factors (peroxisome proliferator-activated receptors) or acting on several membrane receptors (class A scavenger receptors). Heo et al. (89) showed that the use of classic anti-atherosclerotic drugs, such as statins, could promote efferocytosis by activating extracellular signal regulated kinase 5 (ERK5) to polarize macrophages to M2 phenotype. However, some patients experienced adverse reactions due to long-term use of statins, such as hemorrhagic stroke and new-onset diabetes, so it is imperative to find new drugs.

In addition, there was a great deal of evidence that calcium antagonists also had anti-atherosclerotic properties. Ca^2+^ was involved in all steps of phagocytosis, including macrophage migration, macrophage survival, actin polymerization, phagocytic cup formation during phagocytosis, intracellular processing of ACs phagocytosis and secretion of anti-inflammatory cytokines. Thus, Ca^2+^ based therapy was helpful in the attempt to design personalized and targeted drug treatment for patients with atherosclerotic cardiovascular disease (90). Crucially, macrophage metabolism controls the efferocytosis contributing to effective clearance of ACs. Phagocytic cells require intact lipid metabolic pathways to process lipid ingested by apoptotic bodies, and glycolysis within phagocytes contributes to actin polymerization and sustained uptake of corpses (91). However, in addition to glucose and lipid metabolism, Yurdagul et al. (42) found that the continuous efferocytosis of macrophages may affect by amino acid metabolic pathways. Arginine and ornithine of ACs were converted by Arg1 and ODC of macrophages into putrescine, and bone marrow deficient Agr1 or ODC mice had abnormal efferocytosis. Putrescine activated Rac1 to promote the subsequent efferocytosis of macrophages and promote the resolution of AS. As a result, modulation metabolism in macrophages may be a potential therapy for AS.

Besides, the damage of efferocytosis-related signals plays an important role in the development of AS undoubtedly. Wang et al. (92) proposed that the peroxidase proliferation receptor activation receptor could up-regulate the expression of Mertk and play a positive role in phagocytosis in advanced plaques. Luo et al. (93) found that activation of the “Find me” signal S1P attracted macrophages to the position of ACs for their clearance. Additionally, the “Don’t eat me” signal played an extremely important role in efferocytosis therapy. Kojima et al. (7) indicated that CD47 prevented efferocytosis by binding to SIRPα signal receptors in phagocytes. Although the CD47 antibody could promote efferocytosis, the therapy may cause erythrophagocytosis and anemia in some cases. How to improve the recognition and clearance of ACs by phagocytes, to avoid the accidental damage of normal cells is still a concern for this kind of treatment. The above researches have confirmed that enhancing the efferocytosis played a positive role in inhibiting AS and improving the stability of plaque.

## Future Direction

There are many molecules related to the process of efferocytosis in AS lesions, and the over or under expression of any one of the molecules would cause the defect of efferocytosis. Thus, it is essential to determine whether other molecules also operate in efferocytosis. A more comprehensive understanding of the mechanism of AS and the influencing factors related to efferocytosis will enhance the understanding of AS and adopt targeted treatment methods.

Although reducing cell death is likely to decrease AS lesion size, as previously noted, apoptosis in early lesions helped to reduce plaque size, and successful phagocytosis by phagocytes promoted cholesterol efflux and signal generation. Therefore, it is obviously a more effective strategy to enhance phagocytosis by developing efferocytosis signals. As an important “Don’t eat me” molecule, CD47 expressing cells can evade immune surveillance, which was first found in tumor research, and then found to be related to impaired efferocytosis (94). The correlation between AS and tumor pathogenesis has also been paid attention to by researchers, and they seem to have a common pathophysiological substrate (95). Is there a common way for the development of tumor and AS? Further study of the expression of other “Don’t eat me” signals in AS may reveal new therapeutic targets. A significant example that CD24, a “Don’t eat me” signal on tumor cells, was found to be complementary to CD47. Some cancer cells insensitive to CD47 signal blocking turned out to be sensitive to CD24 blocking, such as ovarian cancer (27). According to this point, CD24 as the most promising molecule is expected to be further studied in the treatment of AS. In the future, the application of tumor immune-related therapy to enhance the efferocytosis of AS will be an important research direction. In addition, there has been evidence that many macrophages in AS plaques originated from SMCs. After cholesterol loading, decreased expression of SMC-related genes α-actin, α-tropomyosin, myosin heavy chain, and calponin H1 was detected in mouse aorta, while macrophage-related genes CD68, Mac-2, and ABCA1 were increased. And SMCs also expressed macrophage markers in human coronary artery lesions, especially in about 40% of CD68+ cells expressed SMα-actin in advanced lesion (96). Similar to the apoptosis of macrophages, the apoptosis of SMCs induced the destruction of fibrous plaques (97). However, *in vitro* studies have found that the phagocytic ability of SMC-derived macrophages was lower than that of monocyte-derived macrophages (70). Is the mechanism of impaired efferocytosis in SMC-derived macrophages in AS the same as in classical macrophages? Comparing the difference between them can better understand the plaque environment of AS and adopt targeted treatment strategies. In addition, arteries also contain resident macrophages, but how they functionally differ from recruited macrophages and contribute to plaque macrophage burden is unclear (49). The bulk of macrophages in the plaque is likely derived from blood monocytes recruited, so analysis of the efferocytosis of macrophages from different origins may be helpful for AS prevention.

Moreover, with the popularization of a large number of genetic data sets, regulation of efferocytosis from an epigenetic point of view has recently received much attention. Indeed, macrophage function could be epigenetically regulated, and recent studies have shown that noncoding RNAs as well as DNA methylation regulating efferocytosis may be potential therapeutic targets for AS. Inhibition of highly expressed miRs or replacement of lowly expressed miRs in AS might be a potential therapeutic approach for different diseases. For example, miR-33, an intronic miRs located within the SREBF2 gene, suppresses expression of the cholesterol transporter ABCA1 and lowers HDL levels. Increasing HDL levels with anti-miR33 oligonucleotide treatment promotes reverse cholesterol transport and regression of AS (98). However, some miRs had pleiotropic functions and their therapeutic modulation may lead to unexpected biological effects (99). In addition, a recent report revealed that macrophage function in chronic obstructive pulmonary disease was under epigenetic control of specific target genes by DNA methylation, and reduced methylation of the S1PR5 gene promoter may underlie impaired efferocytosis (100). Especially, it has been indicated that the degree of DNA methylation in ACs played a vital role in controlling the immune response after phagocytosis of ACs. There might be a potential risk of enhancing ACs engulfment in SLE and RA disease, because engulfment of unmethylated DNA favors IL-6 production. The degree of DNA methylation within ACs conferred their immunomodulatory plasticity, and ACs DNA remethylation restored the ability to suppress inflammation (101). Therefore, targeting epigenetic regulation of efferocytosis may be a more fundamental approach, and the study of the efficacy and safety of miR-targeted therapy regimens as well as DNA methylation is of great interest.

Finally, the type, stage, and response of cell death are very important in determining the outcome of AS. In addition to apoptosis, there are many other forms of programmed cell death occur in AS, which promotes the development of AS in some cases, such as pyroptosis, necroptosis, and ferroptosis. Studies targeting these death types may be more effective in reducing plaque formation and rupture. Whereas the ultimate destination of these dead cells is currently less studied, thereby its needs to be further studied in the future. Taken together, the important pathological significance of efferocytosis in AS has been recognized by the academic community. And with the continuous emergence of new therapies targeting to enhance efferocytosis, it may promote the development of anti-atherosclerotic therapy. However, many compounds are still undergoing laboratory studies and tested in animal models or AS patients, so further in-depth study of the detailed mechanism of the above compounds is necessary.

## Conclusion

AS is a systemic chronic inflammatory disease with complicated pathogenesis. Efferocytosis involves the regulation of various molecules to maintain homeostasis, and it is necessary to concentrate on elucidating the signal pathways and factors regulating the process. Evidence is accumulating that the defect of efferocytosis plays an important role in the development of AS. Efferocytosis therapy could provide a new research direction and transformation possible for the prevention and treatment of cardiovascular disease. It has been found that treatments targeting M2 macrophage turnover, activation of Rac1, some non-coding RNA, and enhancement or inhibition of certain efferocytosis signals may break the vicious cycle between efferocytosis and AS. At present, efferocytosis related molecules in the treatment of AS is not comprehensive in human studies, and more extensive and in-depth studies will need to be carried out to further clarify the pathophysiological mechanism of efferocytosis in AS, thereby design new treatments based on efferocytosis mechanism to reduce the risk and complications of clinical AS.

## Author Contributions

All authors contributed to the article and approved the submitted version. LW, HL, YT, and PY conceived and wrote the manuscript.

## Funding

This work was supported in part by the National Natural Science Foundation of China (No. 81973044).

## Conflict of Interest

The authors declare that the research was conducted in the absence of any commercial or financial relationships that could be construed as a potential conflict of interest.
